# Prognostic Impact of *let-7e* MicroRNA and Its Target Genes in Localized High-Risk Intestinal GIST: A Spanish Group for Research on Sarcoma (GEIS) Study

**DOI:** 10.3390/cancers12102979

**Published:** 2020-10-14

**Authors:** Antonio Fernandez-Serra, David S. Moura, María Dolores Sanchez-Izquierdo, Silvia Calabuig-Fariñas, Maria Lopez-Alvarez, Andrea Martínez-Martínez, Irene Carrasco-Garcia, Marta Ramírez-Calvo, Elena Blanco-Alcaina, Raquel López-Reig, Antonia Obrador-Hevia, Regina Alemany, Antonio Gutierrez, Nadia Hindi, Andres Poveda, Jose A. Lopez-Guerrero, Javier Martin-Broto

**Affiliations:** 1 Laboratory of Molecular Biology, Fundación Instituto Valenciano de Oncología, 46009 Valencia, Spain; afernandez@fivo.org (A.F.-S.); anmar59@upv.es (A.M.-M.); mramirezc@fivo.org (M.R.-C.); rlopez@fivo.org (R.L.-R.); 2 Institute of Biomedicine of Sevilla (IBIS, HUVR, CSIC, Universidad de Sevilla), 41013 Sevilla, Spain; david.moura@usal.es (D.S.M.); marlopalv4@gmail.com (M.L.-A.); irenecg1990@gmail.com (I.C.-G.); elena.blancoalcaina@gmail.com (E.B.-A.); nhindi@mustbesevilla.org (N.H.); 3 Instituto de Investigación Sanitaria La Fe, 46026 Valencia, Spain; maria.d.sanchez@uv.es; 4 Molecular Oncology Laboratory, Fundación Investigación, Hospital General Universitario de Valencia, 46014 Valencia, Spain; calabuix_sil@gva.es; 5 Centro de Investigación Biomédica en Red de Cáncer (CIBEROnc), 28029 Madrid, Spain; 6 Department of Pathology, Universitat de València, 46003 Valencia, Spain; 7 Medical Oncology Department, University Hospital Virgen del Rocio, 41013 Sevilla, Spain; 8 Group of Advanced Therapies and Biomarkers in Clinical Oncology, Institut d’Investigació Sanitària de les Illes Balears (IdISBa-IUNICS), 07120 Palma de Mallorca, Spain; antonia.obrador@ssib.es; 9 Sequencing Unit, University Hospital Son Espases, 07120 Palma de Mallorca, Spain; 10 Department of Biology, Balearic Islands University, 07122 Palma de Mallorca, Spain; regina.alemany@uib.es; 11 Hematology Department, University Hospital Son Espases, 07120 Mallorca, Spain; antoniom.gutierrez@ssib.es; 12 Medical Oncology Department, Fundación Instituto Valenciano de Oncología, 46009 Valencia, Spain; apoveda@fivo.org; 13 Department of Basic Medical Sciences, School of Medicine, Catholic University of Valencia ‘San Vicente Martir’, 46001 Valencia, Spain

**Keywords:** GIST, *let-7e*, prognostic biomarkers, caspase-3, *miR-550*

## Abstract

**Simple Summary:**

For intestinal localized high-risk gastrointestinal stromal tumors (GIST) patients’ new molecular biomarkers are urgently needed for a more accurate prognosis. In our study, miRNA profiling analyses was planned to explore new molecular biomarkers with potential prognostic role in this clinical context. Our data, revealed that 39 microRNAs (miRNAs) were significantly deregulated when comparing patients with disease relapsed versus non-relapsed cases. The underexpression of a specific miRNA *let-7e* and the overexpression of 4 of its target genes (*ACVR1B*, *CASP3*, *COL3A1* and *COL5A2*) correlated significantly with worse relapse-free survival. Overall, our results suggest that miRNA profiling is a potential molecular tool useful for a more accurate prognosis for intestinal localized high-risk GIST patients.

**Abstract:**

MicroRNAs (miRNAs) are small non-coding RNAs that negatively regulate gene expression at the post-transcriptional level, and they have been described as being associated with tumor prognosis. Here, miRNA profiling was planned to explore new molecular prognostic biomarkers in localized intestinal high-risk GIST. Paraffin tumor blocks of 14 and 86 patients were used in the discovery and expansion sets, respectively. GeneChip miRNA v3.0 was employed to identify the miRNAs differentially expressed between relapsed and non-relapsed patient samples, which were validated in the expansion set, by qRT-PCR. RT2 Profiler PCR Array was used for the screening of *let-7e* targets. Expression levels were correlated with relapse-free survival and overall survival. In the discovery set, 39 miRNAs were significantly deregulated, *let-7e* and *miR-550* being the most underexpressed and overexpressed miRNAs in the relapsed group, respectively. In the expansion set, the underexpression of *let-7e* or the overexpression of 4 of its target genes (*ACVR1B*, *CASP3*, *COL3A1,* and *COL5A2*) were statistically associated with worse relapse-free survival. The expression of *let-7e* and 4 of its target genes are potential prognostic biomarkers in high-risk localized intestinal GIST. The expression of these genes is a potential molecular tool useful for a more accurate prognosis in this subset of GIST patients.

## 1. Introduction

Gastrointestinal stromal tumors (GISTs) are the most common mesenchymal neoplasms of the gastrointestinal tract. GISTs represent a morphological and biological continuum from incidentally discovered, <10 mm nodules to large sarcomas [1]. GISTs represent a paradigmatic solid-tumor model for targeted therapy with 3 tyrosine-kinase inhibitors (TKI) registered for first [2], second [3] and third [4] lines in advanced disease and adjuvant Imatinib for high-risk localized GIST [5]. Despite this enormous therapeutic investigation on molecular targeted therapy in this entity, the risk classification for localized GIST patients still relies on clinical-pathological features such as mitotic count, size tumor location, and tumor rupture [6]. 

Risk classification of GISTs entails critical information in order to make decisions around adjuvant systemic treatment for these patients. Sometimes, overcounting or undercounting just one mitotic figure can imply 3 years of adjuvant treatment with imatinib. Moreover, those cases for which neoadjuvant treatment is recommended usually have insufficient tumor tissue in the core-biopsy for an adequate mitotic count, making it difficult to establish the risk. In view of the above and in the context of a tumor model for targeted therapies, molecular prognostic variables might be instrumental for a more accurate prognosis delimitation. 

Few studies have explored the prognostic relevance of genomic alterations, suggesting the potential relevance of *KIT* and *PDGFRA* genotyping in prognosis in localized GIST. More specifically, several authors have pointed out the prognostic role of deletion type mutations involving 557 and/or 558 codons of exon 11 in *KIT* [7,8]. Two authors have demonstrated in larger series that it is feasible to incorporate genotype information in risk classification since patients harboring mutations involving 557 and/or 558 had significantly more risk of recurrence in the intermediate subgroup [9,10]. Nevertheless, the prognostic impact of these mutations is restricted only in gastric location. For intestinal localized GIST, no molecular biomarker has been shown to be a convincing prognostic factor. Hence, there is an unmet need for molecular biomarkers for intestinal localized high-risk GIST.

MicroRNAs (miRNAs) encompass a family of more than 2500 small non-coding RNA that negatively regulate gene expression at post-transcriptional level. Based on the stability that they exhibit in formalin-fixed paraffin-embedded (FFPE) blocks [11], miRNAs were found appropriate to be explored in old archival paraffin blocks of GIST, before the adjuvant imatinib time period. With the aim of exploring new molecular biomarkers with a potential prognostic role in localized GIST, a miRNA profiling analysis was planned in the context of intestinal localized high-risk GIST patients. 

## 2. Results

### 2.1. Discovery Set

Fourteen patients, corresponding to cases of high-risk intestinal GIST fitting the inclusion criteria, were analyzed for the miRNA screening. There were 8 cases with relapse and 6 without tumor relapse. The median size was 11 cm (5–25 cm) and the median mitotic count was 12 mitoses per 50 hpf. The demographics of this series is shown in Table 1. 

**Figure 1:**
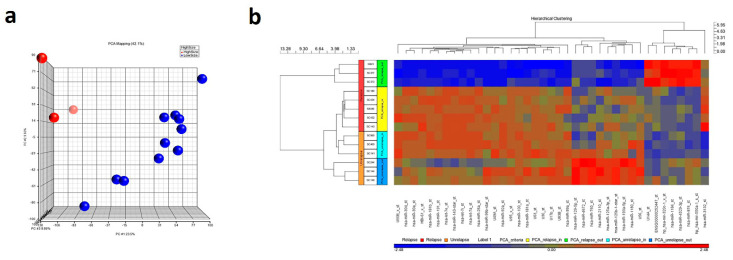
(**a**) Principal component analysis of the 14 samples included in the discovery series. Whole expression of the miRNAs included in the microarray showed a good discriminant power clustering the samples according to its prognosis. (**b**) Clustering and HeatMap considering de-regulated genes in the microarray.

The microRNA expression profiling resulted in 39 miRNAs significantly (*p* ≤ 0.05; FC ≥ 2) deregulated between relapsed and non-relapsed groups. The two main discriminating miRNA between both groups were *lethal-7e* (*let-7e*; underexpressed, FC = −1163.99, *p* < 0.0001) and *miR-550* (overexpressed, FC = 204.173, *p* < 0.0001), when comparing relapsed with non-relapsed patients. Other relevant down-regulated miRNAs in relapsed cases were *miR-17* (FC = −861.879, *p* < 0.0001), *miR-195* (FC = −785.334, *p* < 0.0001) and *miR-143* (FC = −627.526, *p* < 0.0001), while miR-1184 (FC = 184.493, *p* < 0.0001) was up-regulated. However, all those miRNAs presented a lower FC in absolute terms than the selected ones. Within the relapsing cases, the three with the worst prognosis (so-called relapse out group) clustered together (Figure 1).

The prognostic value of both, *let-7e* and *miR-550* was validated by qRT-PCR, confirming its downregulation (FC = 0.6294) and up-regulation (FC = 18.0421), respectively (Figure S1). 

### 2.2. Expansion Set

**Figure 2:**
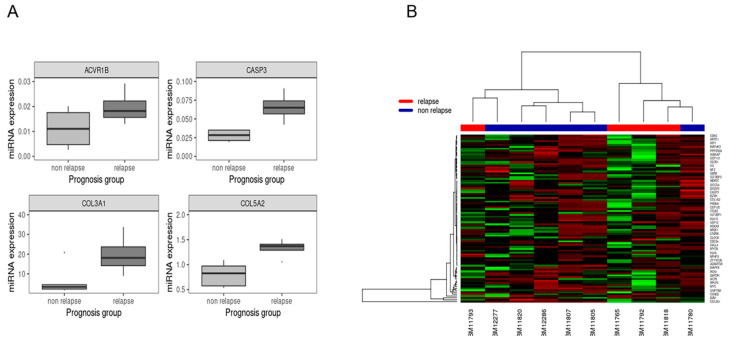
(**A**) Boxplot of the 4 mRNAs showing differential expression between relapse and relapse-free groups. The four genes show significant differential expression: ACVR1B (*p* = 0.0493), CASP3 (*p* = 0.0029), COL3A1 (*p* = 0.039), COL5A2 (*p* = 0.015). (**B**) Heatmap and dendogram of the 84 mRNAs screened as putative targets of *let-7e*.

A subset of 86 localized high-risk intestinal GIST patients, meeting the inclusion criteria, were selected as the expansion set. With a median follow-up of 117 months, 58 patients had a recurrence event (67.4%). Median size and mitoses of the primary tumor were 10 cm and 10/50 hpf, respectively. The actuarial 5-year RFS was 33% in this validation series. The most relevant demographics are depicted in Table 1. The majority of cases were diagnosed with GIST from 1995 to 2000. The univariate analyses for RFS showed statistically significant differences for mitotic count (≤10 vs. >10 mitoses per 50 hpf), tumor location (small intestine vs colorectal), and *let-7e* expression (levels higher or lower than the ROC cutoff). The median RFS was 98.2 (95% CI 0–218.6) and 20 (95% CI 13.5–26.5) months for cases ≤10 and >10 mitoses per 50 hpf, respectively, with *p*-value < 0.001. It was 33.4 (95% CI 23.7–43.1) and 13.4 (95% CI 1.8–25) months for small intestine and colorectal cases respectively with a *p*-value of 0.001, and 41.1 (95% CI 18.2–64) vs. 25 (95% CI 18.2–64) months for cases with *let-7e* expression levels above and below the ROC cutoff, respectively, with a *p*-value of 0.012. The four selected target genes of *let-7e* (Figure 2 and Table S1) showed prognostic relevance in the univariate analysis, following the ROC cutoff. 

Thus, the RFS for values above and below the ROC cutoff were as follows: for *ACVR1B* gene, 28.9 (95% CI 16.8–41.1) months and 43.6 (95% CI 8.1–79.1) months with a *p*-value of 0.023; for the *COL3A1* gene, 21.3 (95% CI 7.5–35.2) months and 39.5 (95% CI 26.9–52.2) months with *p*-value of 0.012; for *COL5A2* gene, 14.1 (95% CI 2.4–25.7) months and 35.5 (95% CI 23.9–47.1) months, with *p*-value of 0.003 and for *CASP3* gene, 26.5 (95% CI 16.1–37) months and 39.5 (95% CI 4–75.1) months with *p*-value 0.018. The complete information of univariate analysis is represented in Table 2. 

**Figure 3:**
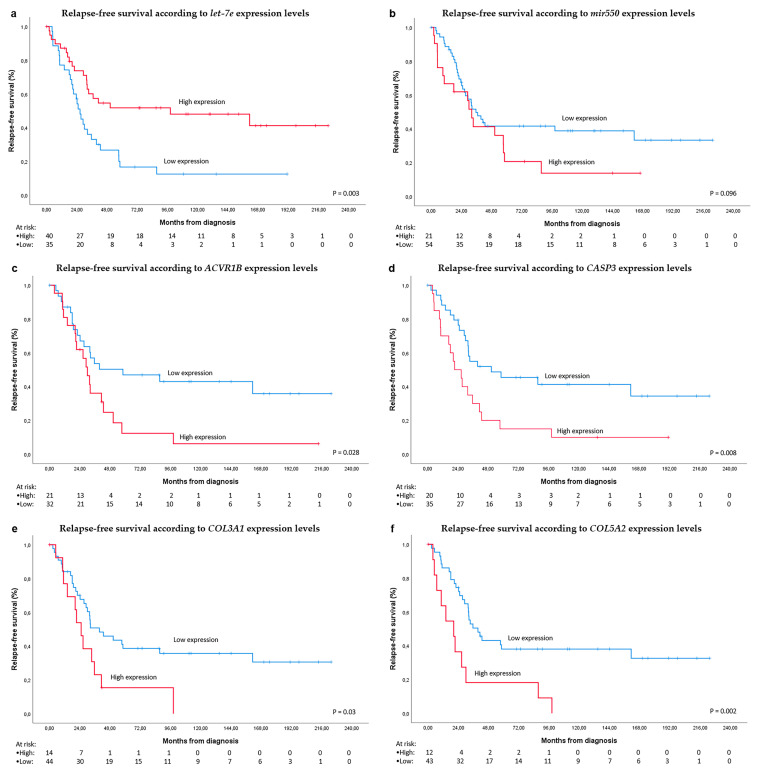
Survival analyses. (**a**) relapse-free survival (RFS) according to *let-7e* expression levels in the small intestine validation set; (**b**). RFS according to *MIR550* expression levels; (**c**) RFS according to *ACVR1B* expression levels; (**d**) RFS according to *CASP3* expression levels; (**e**) RFS according to *COL3A1* expression levels; (**f**) RFS according to *COL5A2* expression levels. Cutoff points were calculated with the ROC curve, separating into High and Low expression groups, when the expression levels were above or below the cutoff, respectively.

Additionally, in order to replicate more precisely the discovery set and to select a more homogeneous population, the analysis was replicated specifically in the 75 small intestine cases. The same prognostic factors in the whole series exhibited a statistically significant difference for RFS in the small intestine series, showing greater differences between prognostic groups (Table 2 and Figure 3). The prognostic role of *miR-550* was not confirmed in the expansion set (Figure S2), neither in the whole series nor in the small intestine subset. In the whole series, cases with *let-7e* overexpression and low expression of each one of the *let-7e* target genes had significantly better RFS (Figure S3).

In the multivariate analysis, mitotic count higher than 10 mitoses per 50 hpf (HR 3.5; 95% CI 1.7–7.1; *p* = 0.001) and *CASP3* mRNA expression above the ROC cutoff (HR 2.5; 95% CI 1.2–4.9; *p* = 0.010) showed independent worse prognosis for RFS. Likely, in the small intestine series these factors were the only independent prognostic variables: mitotic count higher than 10 mitoses per 50 hpf (HR 3.2; 95% CI 1.5–6.7; *p* = 0.002) and *CASP3* mRNA expression above the ROC cutoff (HR 2.6; 95% CI 1.3–5.3; *p* = 0.008). If *let-7e* target genes are not included, then *let-7e* expression (HR 0.4; 95% CI 0.2–0.8; *p* = 0.005) and mitotic count (HR 2.5; 95% CI 1.3–5.1; *p* = 0.01) remained as independent prognostic predictors. The mitotic count did not correlate with *let-7e* expression (*p* = 0.16). 

Only size showed a prognostic role in the univariate analysis for overall survival from the time of diagnosis. Nevertheless, the fact that imatinib has been available since 2002 and some cases had recurrences earlier than this date could induce bias in the overall survival analysis, confirming the use of relapse events as an optimal surrogate for survival in this series. Besides that, a subset of 77 patients with metastatic progression (58 recurred patients from this localized series along with 19 intestinal GIST with metastatic presentation) was considered for a sub-analysis. Among those, there were 56 cases that received imatinib. The univariate analysis screened *let-7e* and gene expression of *let-7e* targets as prognostic factors for progression-free survival (PFS) from the time of imatinib initiation. Patients with *let-7e* expression above and below the ROC cutoff value had progression-free survival of 69.8 (95% CI, 51.3–88.3) and 25.4 months (95% CI, 21.1–29.6), respectively, with a *p*-value of 0.098. Among the analyzed target genes of *let-7e*, *ACVR1B*, *CASP3*, and *COL5A2* showed the same trend observed in the relapse-free survival analysis, a shorter imatinib PFS for values above the ROC cutoff value (Table S2). These differences did not reach statistical significance, probably in relation to the lower number of cases, since only in 33 cases was the RNA expression of these target genes available for the subset with advanced disease treated with imatinib. 

## 3. Discussion

In this high-risk series of localized, completely resected intestinal GIST and imatinib adjuvant naïve, *let-7e* was the most significant under-expressed miRNA in the worse prognostic subset of the discovery set. Its prognostic value was validated in the expansion series, showing an independent prognostic value for RFS. *let-7* constitutes a family of miRNA with 13 members encoding 9 mature miRNA that control developmental timing and differentiation [12]. Even when *let-7* has been described as acting as an oncogene [13], in the vast majority of cases *let-7* members also act as tumor suppressor genes [14,15]. Interestingly, *let-7* is not only found to be decreased in a plethora of tumors, but the underexpression of several members of this family have been associated with poor tumor prognosis [16,17]. Concerning the mechanisms of action linked with prognosis, *let-7* has been involved in several signaling pathways, which could explain its prognostic implication. Suppression of oncogene *HMGA2* [18], inhibition of stemness phenotype [19,20], reduction of phosphorylation of carcinogenic signaling as AKT [21], influence in immunomodulation [22], suppression of proliferation [23,24] and control of invasion/migration [25,26] are among the different mechanisms by which *let-7* can modulate tumor prognosis. 

The screening for putative mRNA targets of *let-7e*, revealed that 4 genes (*ACVR1B, CASP3*, *COL3A1,* and *COL5A2*) had a significant prognostic impact on RFS, in both the discovery and expansion sets. Overexpression of each of these genes was related to underexpression of *let-7e* and with a worse risk of RFS in this intestinal localized GIST series. To the best of our knowledge, this is the first time that *let-7e* and these putative target genes have been related to prognosis in GIST. 

Specifically, *let-7e* has been related to migration and invasion in some tumors. In breast cancer, *let-7e* negatively regulates Myeloid Zinc Finger 1 (MZF1), a transcription factor that ultimately upregulates lysosomal cathepsins B and L in the context of ErbB2 positive breast cancer [27]. In papillary thyroid carcinoma, *let-7e* inhibits migration and invasion through the downregulation of HMGB1 [28]. Similarly, two of the significantly dysregulated *let-7e* target genes in our study, *COL3A1* and *COL5A2*, have relevant roles in migration and invasion, as has been described in some tumors. In bladder carcinoma, *COL3A1* has been implicated in tumor invasion by regulating the MAPK signaling pathway [29], and in nasopharyngeal carcinoma, *COL3A1* was identified as the only target of miR-29b in relation to migration and invasion [30]. Moreover, overexpression of *COL5A2* has been related to worse prognosis with potential association with invasion and dissemination in bladder carcinoma [31], or in osteosarcoma, as an effector of *NKX2-2* [32], among others. 

Overexpression of *ACVR1B*, a key receptor of bone morphogenetic proteins (BMPs) and an important regulator of the BMP/Wnt signaling pathway and, therefore, for cancer stem cells, was associated with clinically aggressive and poor survival in hepatocellular carcinoma [33]. The implication of *ACVR1B* and BMP in cancer has exhibited a dual function according to different cancer cell types or different BMP ligands, promoting or preventing critical cancer hallmarks such as stemness, migration, or proliferation [34]. Protein expression of Caspase-3, either the cleaved fraction or total protein, was associated with poor prognosis in patients with moderately-differentiated carcinoma of the tongue [35]. The expression of *CASP3* was observed to be elevated in some tumors [36] but decreased in others [37], maybe indicating that its expression and its prognostic influence could be largely influenced by tumor and microenvironment context. It could be speculated that caspase-3 overexpression is an epiphenomenon, at least in tumors with substantial apoptotic activity, since dying cells can release mitogens to promote the so-called apoptosis-induced proliferation [38]. However, pro-apoptotic proteins, such as BAX, are downregulated in GIST, while anti-apoptotic proteins, such as BCL2 members and inhibitor of apoptosis (IAP) proteins, are commonly upregulated in GIST [39]. Therefore, it seems that it is not easy to harmonize the finding that *CASP3* overexpression, which is related to worse RFS in localized intestinal GIST, with the inhibition of apoptosis seen in GIST. It could be assumed that *CASP3* failed to become activated, in the GIST context, to participate in the programmed cell death. Nevertheless, in other tumors, the expression level of cleaved caspase 3 did not correlate with spontaneous apoptosis [40], and the overexpression of cleaved caspase 3, not only of the procaspase-3, showed worse disease-free survival [35] Thus, despite the fact that dysregulation of apoptosis is detected in GIST by different means [41,42], it is still not possible to clarify the fine-tuning among different regulators of apoptosis to properly explain the apparent discordance between higher *CASP3* expression and worse prognosis or apoptosis inhibition in the context of localized intestinal GIST patients. Some miRNAs have been associated with apoptosis dysregulation in GIST, with *MIR221/MIR222* having been related to oncogenic GIST development in two of these studies [43,44]. Our group had reported that over-expression of *MIR221/MIR222* was related to worse survival prognosis in a subset of kit negative GIST patients [45]. 

A weakness of this study, in part owing to the old archived samples, is that genotyping was not feasible in up to 14% of the series. The presence of mutations involving codons 557 and/or 558 in exon 11 of the gene *KIT*, did not exhibit prognostic impact in this series. This is in line with a large study that demonstrated these mutations had a prognostic impact only in gastric GISTs [9]. The absence of a functional validation of these results in *GIST* in vitro experiments is another limitation of this study.

Profiling of miRNAs was preferred over other types of profiling, such as mRNA, due to the higher stability shown by miRNAs between paraffin-embedded blocks and fresh tumor-sample analysis. In our series, archived paraffin blocks were used from cases not receiving adjuvant imatinib, meaning that the majority of them were previous to 2005.

## 4. Materials and Methods 

### 4.1. Study Design, Patients, and Samples

Patients with the following inclusion criteria were selected from the GEIS registry: localized intestinal GIST, classified in accordance with Miettinen criteria as high-risk, having not received neo- or adjuvant imatinib, absence of second primary tumor, and confirmation of no tumor abdominal rupture. Procedures were performed in accordance with guidelines established by the hospital’s Ethics Committee and the study was approved by Institutional Review Boards of the university hospitals Virgen Macarena–Virgen del Rocío, on 31 May 2016. 

### 4.2. Discovery Set

#### RNA Isolation and Microarray Analysis

For the miRNA screening, 14 cases corresponding to patients with high-risk intestinal GIST, which met the inclusion criteria, were analyzed. For the screening of miRNA that discriminates between samples of relapsed and non-relapsed high-risk intestinal GIST, a GeneChip miRNA v3.0 (Affymetrix; Santa Clara, CA, USA) was used. One representative formalin-fixed and paraffin-embedded (FFPE) block was identified from each case, and 5 to 10-µm thick sections were obtained for RNA extraction using the miRNeasy FFPE^®^ kit (Qiagen; Hilden, Germany), according to the manufacturer´s instructions. 

RNA purity and concentration were evaluated spectrophotometrically using NanoDrop ND-2000 (ThermoFisher Scientifics; Waltham, MA, USA). The absorbance 260/280 ratio was greater than 1.77 in all cases, whereas the Absorbance 260/230 ratio was between 1.6 and 2.05.

Quality and related size of total and small RNA were assessed by the microfluidics-based platform Agilent 2100 Bioanalyzer (Agilent; Santa Clara, CA, USA) with two chips: Agilent RNA 6000 Nano Kit for total RNA and Agilent Small RNA kit for low molecular weight RNA. In the first approach, the RIN score ranged from 1.90 to 2.50, while the other kit showed RIN values between 2.40 and 2.60. Electropherograms were visualized with the Agilent 2100 Expert software, including data collection, peak detection, and interpretation of the different profiles. All samples showed similar integrity profiles. FFPE extracted RNA has low integrity, ranging from 200 to 600 bp, but it is acceptable for non-coding RNA studies due to their small size. 

For microarray analysis, 600 ng of total RNA was labeled using a FlashTag HSR RNA labeling kit (Genisphere; Hatfield, PA, USA) and hybridized to the GeneChip miRNA 3.0 Array (Affymetrix), following the manufacturer’s instructions. This version contains 19,913 probesets, including 5818 human premature miRNAs, cajal body associated RNA (scaRNA), small nuclear organizers (snoRNA), and mature miRNA. The GeneChip® Scanner 3000 7G System and reagents from Affymetrix were used to hybridize, wash, stain, and scan the arrays. The hypothesis contrasts were made with an ANOVA test and adjusted *p*-value using the false discovery rate adjustment set to FDR <0.5 was used as significant. Normalization and statistical analysis were performed with Partek Genomic Suite 6.6 software (Partek; St. Louis, MO, USA), and technical validation of differentially expressed miRNAs was conducted using qRT-PCR. 

### 4.3. Expansion Set

Selection criteria for the expansion set were the same as described above: intestinal GIST, classified in accordance to Miettinen criteria as high-risk, having not received neo- or adjuvant imatinib, absence of second primary tumor and confirmation of no abdominal tumor rupture. Patients had to be followed-up with scheduled CT scans by physicians of the GEIS network. Sections were obtained for RNA extraction using the RecoverAll Total RNA Extraction kit (Ambion; Austin, TX, USA). The expression of miRNAs was determined by means of qRT-PCR, using specific Taq-Man probes. miRNAs expression levels were categorized as above or below median values. Kaplan–Meier and log-rank tests were used for time-to-event variables with RFS, being the clinical endpoint. 

#### 4.3.1. Quantitative RT-PCR for miRNA Quantification

A biological validation was carried out on the expansion set (*n* = 86). For these cases, one representative block was selected and 3 × 20-μm-thick sections were cut. RNA was isolated using the RecoverAll™ Total Nucleic Acid Isolation Kit for FFPE (Ambion), according to the manufacturer’s instructions. RNA concentration was measured in a NanoDrop-1000 spectrophotometer (ThermoFisher Scientific, Waltham, MA, USA) absorbance 260/280 and 260/230 ratios were measured to assess the quality of the samples.

Reverse transcription was performed from 200 ng of total RNA using the MicroRNA Reverse Transcription Kit^®^ (Applied Biosystems; Foster City, CA, USA), according to the manufacturer´s instructions.

The expression levels of the studied miRNA were determined by means of real-Time PCR using the following TaqMan miRNA assays (Applied Biosystems): *let-7e* (RT:002406), *miR-550* (001544), in a 7500 Fast instrument (Applied Biosystems). RNU-44 (TM1094) and RNU48 (TM1006) were used as housekeeping genes.

#### 4.3.2. Screening of mRNA Targets of *let-7e*

The screening for putative mRNA targets of *let-7e* was performed with a specific RT2 Profiler PCR Array PAHS-6008Y (Qiagen) according to the manufacturer´s instructions. miRNAs were selected by the manufacturer (Qiagen) according to biological or bioinformatic evidence. Besides, mRNA targets have been characterized as *let-7e* targets in public databases such as miR-DB using its own algorithm [46,47]. Each array contains 84 target genes plus 5 housekeeping genes. Briefly, an initial reverse transcription reaction was performed. The quantitative real-time PCR interrogating 84 genes previously validated as human targets of *let-7* was used (Appendix A). This screening was carried out in ten samples previously used in the biological validation of the differentially-expressed miRNA expression, of which 4 correspond to relapsing samples. For this analysis, samples with optimum quality were chosen from among the biological validation set.

#### 4.3.3. Quantitative RT-PCR for the Validation of mRNA Targets

**Figure 4:**
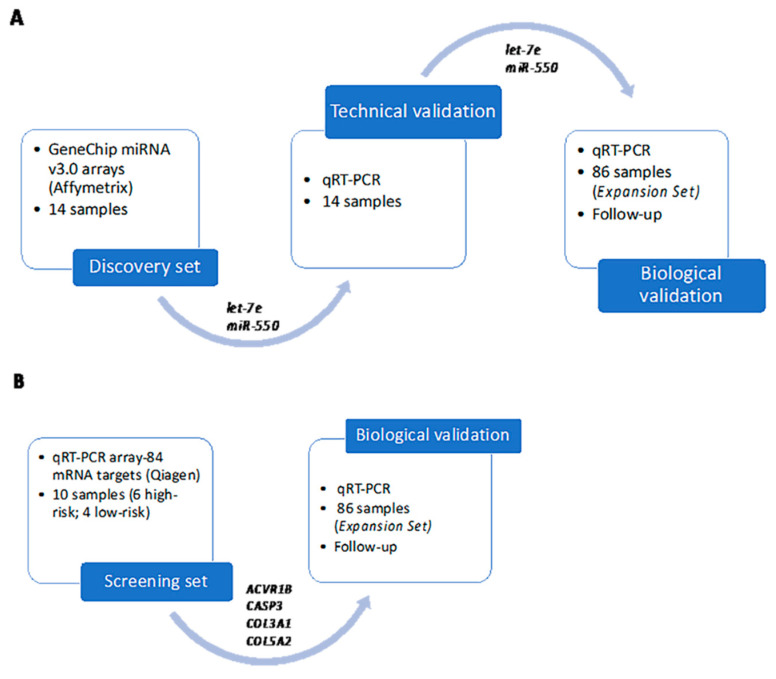
(**A**) study design to discover miRNAs differentially expressed with their technical and biological validation; (**B**) screening approach to demonstrate the clinical relevance of potential mRNA-*let-7e* targets. KIT and PDGFRA genotyping.

The expression levels of the genes selected in the mRNA target screening of *let-7* were measured by means of qRT-PCR using the following TaqMan RNA assays (Applied Biosystems): *ACVR1B* (Hs00244715_m1), *CASP3* (Hs00234387_m1), *COL3A1* (Hs00943809_m1), and *COL5A2* (Hs00893878_m1). While *B2M* (Beta-2-microglobulin; Hs99999907_m1) and *GAPDH* (Hs00266705_g1) were used as housekeeping genes for data normalization. A 7500 Fast instrument (Applied Biosystems) was used. After data normalization, the relative expression of the genes was evaluated, applying the 2^−ΔΔCt^ method for the Fold Change (FC) calculation. Figure 4 outlines all the above-mentioned steps in search of biomarkers in this study.

Among the 86 cases, 50 were sequenced using Sanger sequencing and the rest of the cases with NGS. DNA was isolated from 3- to 5-μm sections of FFPE tissues. After deparaffinization, the tumor tissue was processed with the QIAamp DNA Investigator Kit (Qiagen) according to the manufacturer´s instructions. Intronic primers were used to amplify exons 9, 11, 13 [48] and 17 [49] of *KIT* and exons 12 and 18 of *PDGFRA* [50] by PCR, as previously described in Martin et al. [8]. PCR products were bi-directionally sequenced in F and R, with the specific intronic primers used in the amplification diluted to a work concentration of 3.2 μM. Sequencing was performed in an ABI 3130xl sequencer using the BigDye Terminator v3.1 (Applied Biosystems).

*KIT* and *PDGFRA* genotyping, by NGS, was performed using the GIST MASTR kit, following manufacturer’s instructions (Multiplicom; Niel, Belgium). The assay generates a library of specific amplicons in two rounds of PCR followed by purification using Agencourt AMPure XP (Beckman Coulter, Beverly, MA, USA) and quantitation with PicoGreen, as described in Feliubadalo et al. [51].

Aligned sequences were visualized with Integrative Genomic Viewer Software 2.4.8 [52], and Variant calling was analyzed using Variant Studio 2.2 (Illumina; San Diego, CA, USA).

#### 4.3.4. Statistical Analysis

Data analyses were performed with R v3.3.1 using R studio 0.99.486 and SPSS version 26 (IBM; Chicago, IL, USA). For categorical variables, frequency and percentages were calculated. For gene expression categorization, we calculate the optimal cutoff using ROC curves for their impact on progression and death. For time-to-event variables (i.e., relapse-free survival (RFS) and overall survival (OS)), we used Kaplan-Meier plots with a log-rank test to compare groups. For multivariate survival analysis, we implemented multivariate analysis with the variables that appeared to be significant in the univariate analysis, and the test was carried out according to the Cox proportional-hazard regression model with a backward variable selection. Linear regression was performed to correlate quantitative variables. All *p*-values reported were two-sided, and statistical significance was defined at *p* < 0.05.

#### 4.3.5. Data Availability

Raw data from microarray experiments are available in Gene Expression Omnibus (GEO) from NCBI: GSE156715156715.

## 5. Conclusions

In conclusion, our analysis from genomic profiling of miRNAs, in a homogeneous high-risk population of localized intestinal GIST, indicated the potential prognostic relevance in RFS for *let-7e* and 4 of its target genes: *ACVR1B, CASP3*, *COL3A1,* and *COL5A2*. This knowledge opens new options for prognostic biomarkers and potential therapeutic targets. The mechanistic link underlying their prognostic role is currently being investigated in our laboratory. 

## Figures and Tables

**Table 1:** Series demographics.

Clinical and Pathological Parameters	Discovery Set *N* = 14	Validation Set *N* = 86	Validation Set Small-Intestine Series *N* = 75
**Age: median (range)**	69 (56–87)	62 (33–87)	62 (33–87)
**Sex: male/female (%)**	7 (50%)/7 (50%)	52 (61%)/34 (39%)	47 (63%)/28 (37%)
**Median follow–up (months)**	85 (0–198)	73 (0–253)	76 (0–253)
**Median size of primary tumors (cm; range)**	11 (5–25)	10 (4–33)	10 (4–33)
**Median mitotic count (/50 HPF) (range)**	13 (0–133)	10 (0–133)	10 (0–133)
**Primary tumor site:**			
**Small–intestine (%)**	14 (100%)	75 (87%)	75 (100%)
**Rectum (%)**	0 (0%)	4 (5%)	0 (0%)
**Colon (%)**	0 (0%)	1 (1%)	0 (0%)
**Omentum (%)**	0 (0%)	1 (1%)	0 (0%)
**Peritoneum**	0 (0%)	3 (4%)	0 (0%)
**Others**	0 (0%)	2 (2%)	0 (0%)
**Relapse:**			
- **Yes** - **No**	8 (58%) 6 (42%)	58 (67%) 28 (330%)	49 (65%) 26 (35%)
**Genotype:**			
- **Wild type** - ***KIT* mutation** - ***PDGFRA* mutation** - **Not available**	4 (29%) 9 (64%) 1 (7%) 0 (0%)	18 (20%) 52 (61%) 4 (5%) 12 (14%)	17 (23%) 47 (63%) 2 (2%) 9 (12%)
***KIT* mutation:**			
- **Exon 11** - **Exon 9** - **Exon 13**	6 (67%) 2 (22%) 1 (11%)	44 (85%) 7 (14%) 1 (1%)	40 (85%) 6 (13%) 1 (2%)
**Exon 11 mutation:**			
- **Affecting codon 557/558** - **Not affecting codon 557/558**	3 (50%) 3 (50%)	17 (39%) 27 (61%)	17 (43%) 23 (57%)

**Table 2:** Univariate analyses.

Factor	Whole Series			
	Median RFS (CI 95%)	*p*	Median OS (CI 95%)	*p*
Age:		0.34		0.4
- 0–60 - >60	26.5 (15.1–37.9) 31.7 (22.7–40.8)		155 (*N*A) *N*R	
Sex:		0.61		0.62
- Male - Female	31.7 (24.6–38.8) 32.2 (4.5–59.8)		155.9 (43.4–268.4) *N*R	
Size:		0.075		0.001
- 0–10 - >10	32.2 (15.8–48.5) 27.3 (13.3–41.2)		*N*R 73.4 (52.9–93.9)	
Mitosis:		<0.001		0.13
- 0–10 - >10	98.2 (0–218.6) 20 (13.5–26.5)		*N*R 78 (37.7–118.2)	
Critical mutation:		0.64		0.8
- Yes - No	32.2 (16.2–48.1) 31.7 (21.4–42)		*N*R 155.9 (36.7–275.2)	
Tumor location:		0.001		0.22
- Small-intestine - Other	33.4 (23.7–43.1) 13.4 (1.8–25)		155.9 (*N*A) 77.6 (16.2–139.1)	
*let-7e* expression *:		0.012		0.32
- <131.42 - >131.42	25 (18.2–31.8) 41.1 (18.2–64)		90.2 (73.5–106.9) *N*R	
*miR550* expression *:		0.136		0.55
- <4063.67 - >4063.67	31.9 (21.1–42.6) 29.9 (20.7–39.2)		155.9 (*N*A) NR	
*ACVR1B* expression *:		0.023		0.54
- <0.000629 - >0.000629	43.6 (8.1–79.1) 28.9 (16.8–41.1)		*N*R 117.3 (42.4–192.2)	
*CASP3* expression *:		0.018		0.71
- <0.000769 - >0.000769	39.5 (4–75.1) 26.5 (16.1–37)		*N*R 155.9 (23.2–288.6)	
*COL3A1* expression *:		0.012		0.037
- <0.671380 - >0.671380	39.5 (26.9–52.2) 21.3 (7.5–35.2)		*N*R 61.8 (23.9–99.7)	
*COL5A2* expression *:		0.003		0.26
- <0.014649 - >0.014649	35.5 (23.9–47.1) 14.1 (2.4–25.7)		*N*R 81.1 (0–167.6)	
	**Median RFS** **(CI 95%)**	***p***	**Median OS** **(CI 95%)**	***p***
Age:		0.38		0.76
- 0–60 - >60	32.4 (15.1–49.6) 35.5 (6.2–64.9)		155 (*N*A) *N*R	
Sex:		0.5		0.75
- Male - Female	31.9 (24.3–39.6) 50.5 (9.8–91.1)		155.9 (22.7–289.1) *N*R	
Size:		0.061		0.005
- 0–10 - >10	36.9 (5.5–68.3) 28.9 (11.4–46.5)		*N*R 73.4 (49.4–97.4)	
Mitosis:		<0.001		0.14
- 0–10 - >10	161.2 (2.7–319.6) 20.8 (15.4–26.1)		*N*R 90.2 (36.3–144)	
Critical mutation:		0.22		0.63
- Yes - No	32.4 (10.8–53.9) 32.2 (16.2–48.1)		*N*R 155.9 (36.7–275.2)	
Tumor location:				
- Small-intestine - Other	*N*A		*N*A	
*Let-7e* expression *:		0.003		0.38
- <131.42 - >131.42	26.5 (20.6–32.5) 98.2 (0–225.1)		90.3 (*N*A) *N*R	
*miR550* expression *:		0.096		0.46
- <4063.67 - >4063.67	35.5 (24.8–46.3) 32.2 (25.9–38.4)		155.9 (*N*A) *N*R	
*ACVR1B* expression *:		0.028		0.57
- <0.000629 - >0.000629	58.2 (0–128.6) 30 (22.7–37.2)		*N*R 155.9 (50.7–261.1)	
*CASP3* expression *:		0.008		0.34
- <0.000769 - >0.000769	50.5 (0–106.7) 21.3 (7.6–35.1)		*N*R 72.1 (0–150.7)	
*COL3A1* expression *:		0.03		0.053
- <0.671380 - >0.671380	39.5 (16.4–62.7) 25 (17.7–32.4)		*N*R 61.8 (30.2–93.5)	
*COL5A2* expression *:		0.002		0.17
- <0.014649 - >0.014649	39.5 (26.6–52.4) 20.3 (8.4–32.1)		*N*R 81.1 (0–168.9)	

* Optimal cutoff calculated using ROC curves.

## Supplementary Materials

The following are available online at https://www.mdpi.com/2072-6694/12/10/2979/s1, Table S1: Results of the mRNA differentially expressed in the target screening series. Table S2: Univariate analysis of metastatic cases treated with Imatinib (*n* = 56). Figure S1: Target miRNA expression in technical validation; Figure S2: Target miRNA expression in biological validation; Figure S3: Relapse-free survival according to *let-7e*.

## Appendix A. mRNA Targets of *let-7e*

NM_004302 ACVR1B Activin A receptor, type IB
NM_007037 ADAMTS8 ADAM metallopeptidase with thrombospondin type 1 motif, 8
NM_138578 BCL2L1 BCL2-like 1
NM_001728 BSG Basigin (OK blood group)
NM_014670 BZW1 Basic leucine zipper and W2 domains 1
NM_004346 CASP3 Caspase 3, apoptosis-related cysteine peptidase
NM_001237 CCNA2 Cyclin A2
NM_053056 CCND1 Cyclin D1
NM_001759 CCND2 Cyclin D2
NM_014711 CCP110 Centriolar coiled coil protein 110kDa
NM_001789 CDC25A Cell division cycle 25 homolog A (S. pombe)
NM_004359 CDC34 Cell division cycle 34 homolog (S. cerevisiae)
NM_001259 CDK6 Cyclin-dependent kinase 6
NM_025009 CEP135 Centrosomal protein 135kDa
NM_001273 CHD4 Chromodomain helicase DNA binding protein 4
NM_004898 CLOCK Clock homolog (mouse)
NM_000089 COL1A2 Collagen, type I, alpha 2
NM_000090 COL3A1 Collagen, type III, alpha 1
NM_000393 COL5A2 Collagen, type V, alpha 2
NM_007242 DDX19B DEAD (Asp-Glu-Ala-As) box polypeptide 19B
NM_177438 DICER1 Dicer 1, ribonuclease type III
NM_004417 DUSP1 Dual specificity phosphatase 1
NM_005225 E2F1 E2F transcription factor 1
NM_004091 E2F2 E2F transcription factor 2
NM_017629 AGO4 Eukaryotic translation initiation factor 2C, 4
NM_015123 FRMD4B FERM domain containing 4B
NM_001482 GATM Glycine amidinotransferase (L-arginine:glycine amidinotransferase)
NM_001001557 GDF6 Growth differentiation factor 6
NM_024312 GNPTAB N-acetylglucosamine-1-phosphate transferase, alpha and beta subunits
NM_017902 HIF1AN Hypoxia inducible factor 1, alpha subunit inhibitor
NM_005338 HIP1 Huntingtin interacting protein 1
NM_152739 HOXA9 Homeobox A9
NM_002176 IFNB1 Interferon, beta 1, fibroblast
NM_006546 IGF2BP1 Insulin-like growth factor 2 mRNA binding protein 1
NM_006547 IGF2BP3 Insulin-like growth factor 2 mRNA binding protein 3
NM_003640 IKBKAP Inhibitor of kappa light polypeptide gene enhancer in B-cells, kinase complex-associated protein
NM_002188 IL13 Interleukin 13
NM_000600 IL6 Interleukin 6 (interferon, beta 2)
NM_001023570 IQCB1 IQ motif containing B1
NM_000212 ITGB3 Integrin, beta 3 (platelet glycoprotein IIIa, antigen CD61)
NM_002776 KLK10 Kallikrein-related peptidase 10
NM_002774 KLK6 Kallikrein-related peptidase 6
NM_004985 KRAS V-Ki-ras2 Kirsten rat sarcoma viral oncogene homolog
NM_024674 LIN28A Lin-28 homolog A (C. elegans)
NM_014813 LRIG2 Leucine-rich repeats and immunoglobulin-like domains 2
NM_153377 LRIG3 Leucine-rich repeats and immunoglobulin-like domains 3
NM_003618 MAP4K3 Mitogen-activated protein kinase kinase kinase kinase 3
NM_001315 MAPK14 Mitogen-activated protein kinase 14
NM_002748 MAPK6 Mitogen-activated protein kinase 6
NM_002750 MAPK8 Mitogen-activated protein kinase 8
NM_002467 MYC V-myc myelocytomatosis viral oncogene homolog (avian)
NM_005378 MYCN V-myc myelocytomatosis viral related oncogene, neuroblastoma derived (avian)
NM_181659 NCOA3 Nuclear receptor coactivator 3
NM_000268 NF2 Neurofibromin 2 (merlin)
NM_017595 NKIRAS2 NFKB inhibitor interacting Ras-like 2
NM_153240 NPHP3 Nephronophthisis 3 (adolescent)
NM_003269 NR2E1 Nuclear receptor subfamily 2, group E, member 1
NM_002524 NRAS Neuroblastoma RAS viral (v-ras) oncogene homolog
NM_015393 PARM1 Prostate androgen-regulated mucin-like protein 1
NM_006195 PBX3 Pre-B-cell leukemia homeobox 3
NM_017990 PDPR Pyruvate dehydrogenase phosphatase regulatory subunit
NM_015715 PLA2G3 Phospholipase A2, group III
NM_005761 PLXNC1 Plexin C1
NM_002717 PPP2R2A Protein phosphatase 2, regulatory subunit B, alpha
NM_182907 PRDM1 PR domain containing 1, with ZNF domain
NM_006267 RANBP2 RAN binding protein 2
NM_006909 RASGRF2 Ras protein-specific guanine nucleotide-releasing factor 2
NM_018211 RAVER2 Ribonucleoprotein, PTB-binding 2
NM_002901 RCN1 Reticulocalbin 1, EF-hand calcium binding domain
NM_020436 SALL4 Sal-like 4 (Drosophila)
NM_000617 SLC11A2 Solute carrier family 11 (proton-coupled divalent metal ion transporters), member 2
NM_006306 SMC1A Structural maintenance of chromosomes 1A
NM_080867 SOCS4 Suppressor of cytokine signaling 4
NM_004612 TGFBR1 Transforming growth factor, beta receptor 1
NM_138554 TLR4 Toll-like receptor 4
NM_001039111 TRIM71 Tripartite motif containing 71
NM_007275 TUSC2 Tumor suppressor candidate 2
NM_152896 UHRF2 Ubiquitin-like with PHD and ring finger domains 2
NM_182488 USP12 Ubiquitin specific peptidase 12
NM_032582 USP32 Ubiquitin specific peptidase 32
NM_014023 WDR37 WD repeat domain 37
NM_005430 WNT1 Wingless-type MMTV integration site family, member 1
NM_014872 ZBTB5 Zinc finger and BTB domain containing 5
NM_015346 ZFYVE26 Zinc finger, FYVE domain containing 26
NM_001101 ACTB Actin, beta
NM_004048 B2M Beta-2-microglobulin
NM_002046 GAPDH Glyceraldehyde-3-phosphate dehydrogenase
NM_000194 HPRT1 Hypoxanthine phosphoribosyltransferase 1
NM_001002 RPLP0 Ribosomal protein, large, P0
SA_00105 HGDC Human Genomic DNA Contamination
SA_00104 RTC Reverse Transcription Control
SA_00104 RTC Reverse Transcription Control
SA_00104 RTC Reverse Transcription Control
SA_00103 PPC Positive PCR Control
SA_00103 PPC Positive PCR Control
SA_00103 PPC Positive PCR Control
