# Supplementary material for: Prognostic Impact of let-7e MicroRNA and Its Target Genes in Localized High-Risk Intestinal GIST: A Spanish Group for Research on Sarcoma (GEIS) Study
Source: Cancers (Basel). 2020 Oct 14;12(10):2979. doi: 10.3390/cancers12102979 (PMC7602387; doi:10.3390/cancers12102979)
Supplement: Supplementary file 1 [file cancers-12-02979-s001.zip › Appendix A.docx]

**mRNA targets of *let-7e:***

NM_004302 ACVR1B Activin A receptor, type IB

NM_007037 ADAMTS8 ADAM metallopeptidase with thrombospondin type 1 motif, 8

NM_138578 BCL2L1 BCL2-like 1

NM_001728 BSG Basigin (OK blood group)

NM_014670 BZW1 Basic leucine zipper and W2 domains 1

NM_004346 CASP3 Caspase 3, apoptosis-related cysteine peptidase

NM_001237 CCNA2 Cyclin A2

NM_053056 CCND1 Cyclin D1

NM_001759 CCND2 Cyclin D2

NM_014711 CCP110 Centriolar coiled coil protein 110kDa

NM_001789 CDC25A Cell division cycle 25 homolog A (S. pombe)

NM_004359 CDC34 Cell division cycle 34 homolog (S. cerevisiae)

NM_001259 CDK6 Cyclin-dependent kinase 6

NM_025009 CEP135 Centrosomal protein 135kDa

NM_001273 CHD4 Chromodomain helicase DNA binding protein 4

NM_004898 CLOCK Clock homolog (mouse)

NM_000089 COL1A2 Collagen, type I, alpha 2

NM_000090 COL3A1 Collagen, type III, alpha 1

NM_000393 COL5A2 Collagen, type V, alpha 2

NM_007242 DDX19B DEAD (Asp-Glu-Ala-As) box polypeptide 19B

NM_177438 DICER1 Dicer 1, ribonuclease type III

NM_004417 DUSP1 Dual specificity phosphatase 1

NM_005225 E2F1 E2F transcription factor 1

NM_004091 E2F2 E2F transcription factor 2

NM_017629 AGO4 Eukaryotic translation initiation factor 2C, 4

NM_015123 FRMD4B FERM domain containing 4B

NM_001482 GATM Glycine amidinotransferase (L-arginine:glycine amidinotransferase)

NM_001001557 GDF6 Growth differentiation factor 6

NM_024312 GNPTAB N-acetylglucosamine-1-phosphate transferase, alpha and beta subunits

NM_017902 HIF1AN Hypoxia inducible factor 1, alpha subunit inhibitor

NM_005338 HIP1 Huntingtin interacting protein 1

NM_152739 HOXA9 Homeobox A9

NM_002176 IFNB1 Interferon, beta 1, fibroblast

NM_006546 IGF2BP1 Insulin-like growth factor 2 mRNA binding protein 1

NM_006547 IGF2BP3 Insulin-like growth factor 2 mRNA binding protein 3

NM_003640 IKBKAP Inhibitor of kappa light polypeptide gene enhancer in B-cells, kinase complex-associated protein

NM_002188 IL13 Interleukin 13

NM_000600 IL6 Interleukin 6 (interferon, beta 2)

NM_001023570 IQCB1 IQ motif containing B1

NM_000212 ITGB3 Integrin, beta 3 (platelet glycoprotein IIIa, antigen CD61)

NM_002776 KLK10 Kallikrein-related peptidase 10

NM_002774 KLK6 Kallikrein-related peptidase 6

NM_004985 KRAS V-Ki-ras2 Kirsten rat sarcoma viral oncogene homolog

NM_024674 LIN28A Lin-28 homolog A (C. elegans)

NM_014813 LRIG2 Leucine-rich repeats and immunoglobulin-like domains 2

NM_153377 LRIG3 Leucine-rich repeats and immunoglobulin-like domains 3

NM_003618 MAP4K3 Mitogen-activated protein kinase kinase kinase kinase 3

NM_001315 MAPK14 Mitogen-activated protein kinase 14

NM_002748 MAPK6 Mitogen-activated protein kinase 6

NM_002750 MAPK8 Mitogen-activated protein kinase 8

NM_002467 MYC V-myc myelocytomatosis viral oncogene homolog (avian)

NM_005378 MYCN V-myc myelocytomatosis viral related oncogene, neuroblastoma derived (avian)

NM_181659 NCOA3 Nuclear receptor coactivator 3

NM_000268 NF2 Neurofibromin 2 (merlin)

NM_017595 NKIRAS2 NFKB inhibitor interacting Ras-like 2

NM_153240 NPHP3 Nephronophthisis 3 (adolescent)

NM_003269 NR2E1 Nuclear receptor subfamily 2, group E, member 1

NM_002524 NRAS Neuroblastoma RAS viral (v-ras) oncogene homolog

NM_015393 PARM1 Prostate androgen-regulated mucin-like protein 1

NM_006195 PBX3 Pre-B-cell leukemia homeobox 3

NM_017990 PDPR Pyruvate dehydrogenase phosphatase regulatory subunit

NM_015715 PLA2G3 Phospholipase A2, group III

NM_005761 PLXNC1 Plexin C1

NM_002717 PPP2R2A Protein phosphatase 2, regulatory subunit B, alpha

NM_182907 PRDM1 PR domain containing 1, with ZNF domain

NM_006267 RANBP2 RAN binding protein 2

NM_006909 RASGRF2 Ras protein-specific guanine nucleotide-releasing factor 2

NM_018211 RAVER2 Ribonucleoprotein, PTB-binding 2

NM_002901 RCN1 Reticulocalbin 1, EF-hand calcium binding domain

NM_020436 SALL4 Sal-like 4 (Drosophila)

NM_000617 SLC11A2 Solute carrier family 11 (proton-coupled divalent metal ion transporters), member 2

NM_006306 SMC1A Structural maintenance of chromosomes 1A

NM_080867 SOCS4 Suppressor of cytokine signaling 4

NM_004612 TGFBR1 Transforming growth factor, beta receptor 1

NM_138554 TLR4 Toll-like receptor 4

NM_001039111 TRIM71 Tripartite motif containing 71

NM_007275 TUSC2 Tumor suppressor candidate 2

NM_152896 UHRF2 Ubiquitin-like with PHD and ring finger domains 2

NM_182488 USP12 Ubiquitin specific peptidase 12

NM_032582 USP32 Ubiquitin specific peptidase 32

NM_014023 WDR37 WD repeat domain 37

NM_005430 WNT1 Wingless-type MMTV integration site family, member 1

NM_014872 ZBTB5 Zinc finger and BTB domain containing 5

NM_015346 ZFYVE26 Zinc finger, FYVE domain containing 26

NM_001101 ACTB Actin, beta

NM_004048 B2M Beta-2-microglobulin

NM_002046 GAPDH Glyceraldehyde-3-phosphate dehydrogenase

NM_000194 HPRT1 Hypoxanthine phosphoribosyltransferase 1

NM_001002 RPLP0 Ribosomal protein, large, P0

SA_00105 HGDC Human Genomic DNA Contamination

SA_00104 RTC Reverse Transcription Control

SA_00104 RTC Reverse Transcription Control

SA_00104 RTC Reverse Transcription Control

SA_00103 PPC Positive PCR Control

SA_00103 PPC Positive PCR Control

SA_00103 PPC Positive PCR Control
