# Supplementary material for: Prognostic Impact of let-7e MicroRNA and Its Target Genes in Localized High-Risk Intestinal GIST: A Spanish Group for Research on Sarcoma (GEIS) Study
Source: Cancers (Basel). 2020 Oct 14;12(10):2979. doi: 10.3390/cancers12102979 (PMC7602387; doi:10.3390/cancers12102979)
Supplement: Supplementary file 1 [file cancers-12-02979-s001.zip › Table S1.docx]

Table S1 - Results of the mRNA differentially expressed in the target screening series.

| **Gene** | **Fold Change** | **p-value** |
| --- | --- | --- |
| *CASP3* | 6.93 | 0.002921 |
| *COL5A2* | 5.97 | 0.015152 |
| *ACVR1B* | 4.97 | 0.049363 |
| *COL3A1* | 4.44 | 0.039564 |
