# Supplementary material for: Prognostic Impact of let-7e MicroRNA and Its Target Genes in Localized High-Risk Intestinal GIST: A Spanish Group for Research on Sarcoma (GEIS) Study
Source: Cancers (Basel). 2020 Oct 14;12(10):2979. doi: 10.3390/cancers12102979 (PMC7602387; doi:10.3390/cancers12102979)
Supplement: Supplementary file 1 [file cancers-12-02979-s001.zip › Table S2.docx]

Table S2 – Univariate analysis of metastatic cases treated with Imatinib (n=56)

|  | Median RFS (95% CI) | p |
| --- | --- | --- |
| *let-7e* expression*:   - < 131.42 - > 131.42 | 25.4 (21.1-29.6)  69.8 (51.3-88.3) | 0.098 |
| *miR550* expression*:   - < 4063.67 - > 4063.67 | 36.9 (19.9-53.8)  33.9 (16.3-51.5) | 0.39 |
| *ACVR1B* expression*:   - < 0.000629 - > 0.000629 | 58.2 (0-128.6)  30 (22.7-37.2) | 0.67 |
| *CASP3* expression*:   - < 0.000769 - > 0.000769 | 60.9 (1.1-120.6)  34.7 (13.3-56.1) | 0.96 |
| *COL3A1* expression*:   - < 0.671380 - > 0.671380 | 34.7 (7.1-62.3)  53.1 (0-115.5) | 0.57 |
| *COL5A2* expression*:   - < 0.014649 - > 0.014649 | 60.9 (19.5-102.2)  15.6 (11.3-20) | 0.21 |

*Optimal cut-off calculated using ROC curves
