# Supplementary figures and images for: Prognostic Impact of let-7e MicroRNA and Its Target Genes in Localized High-Risk Intestinal GIST: A Spanish Group for Research on Sarcoma (GEIS) Study
Source: Cancers (Basel). 2020 Oct 14;12(10):2979. doi: 10.3390/cancers12102979 (PMC7602387; doi:10.3390/cancers12102979)

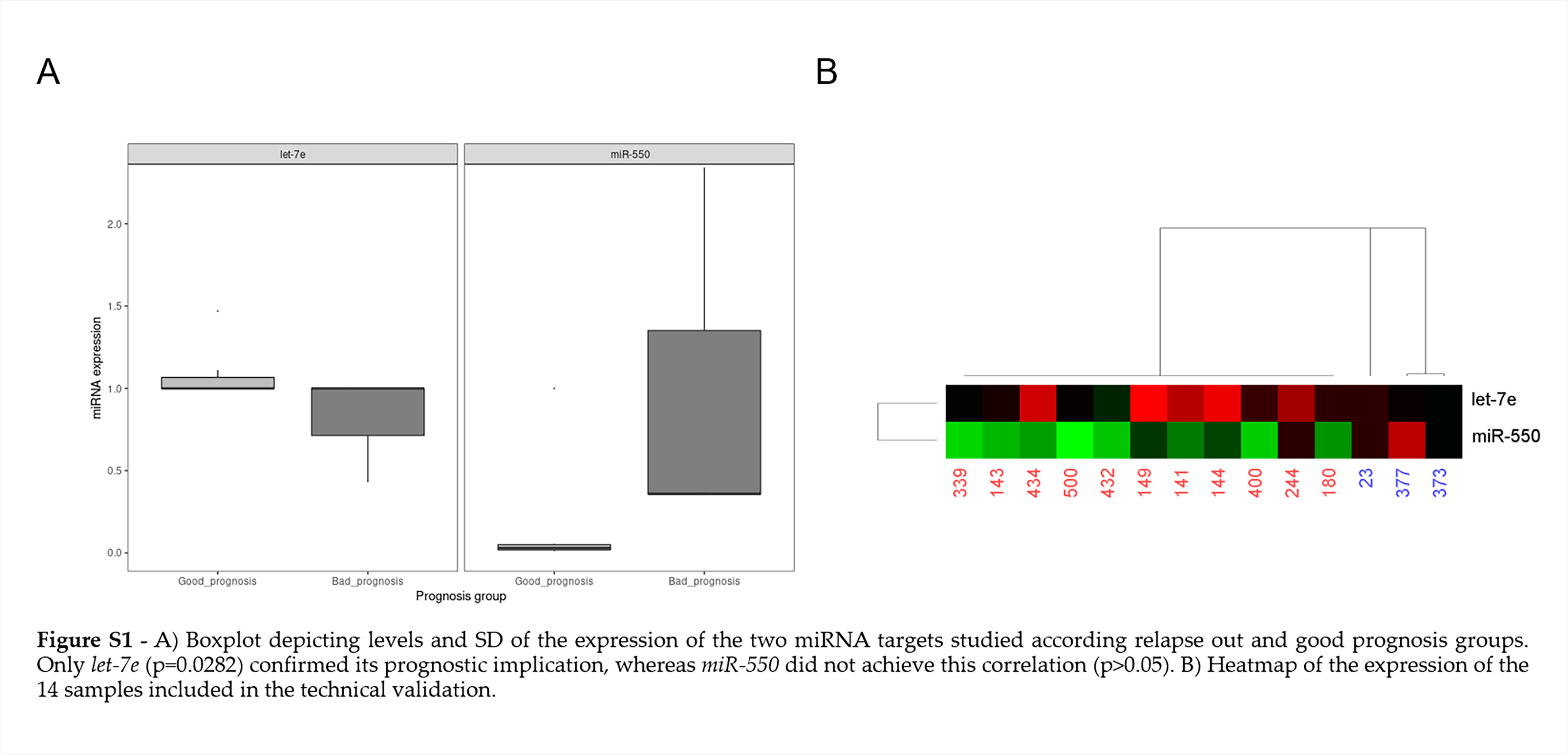

Supplement: Supplementary file 1 [file cancers-12-02979-s001.zip › Figure S1.tif]

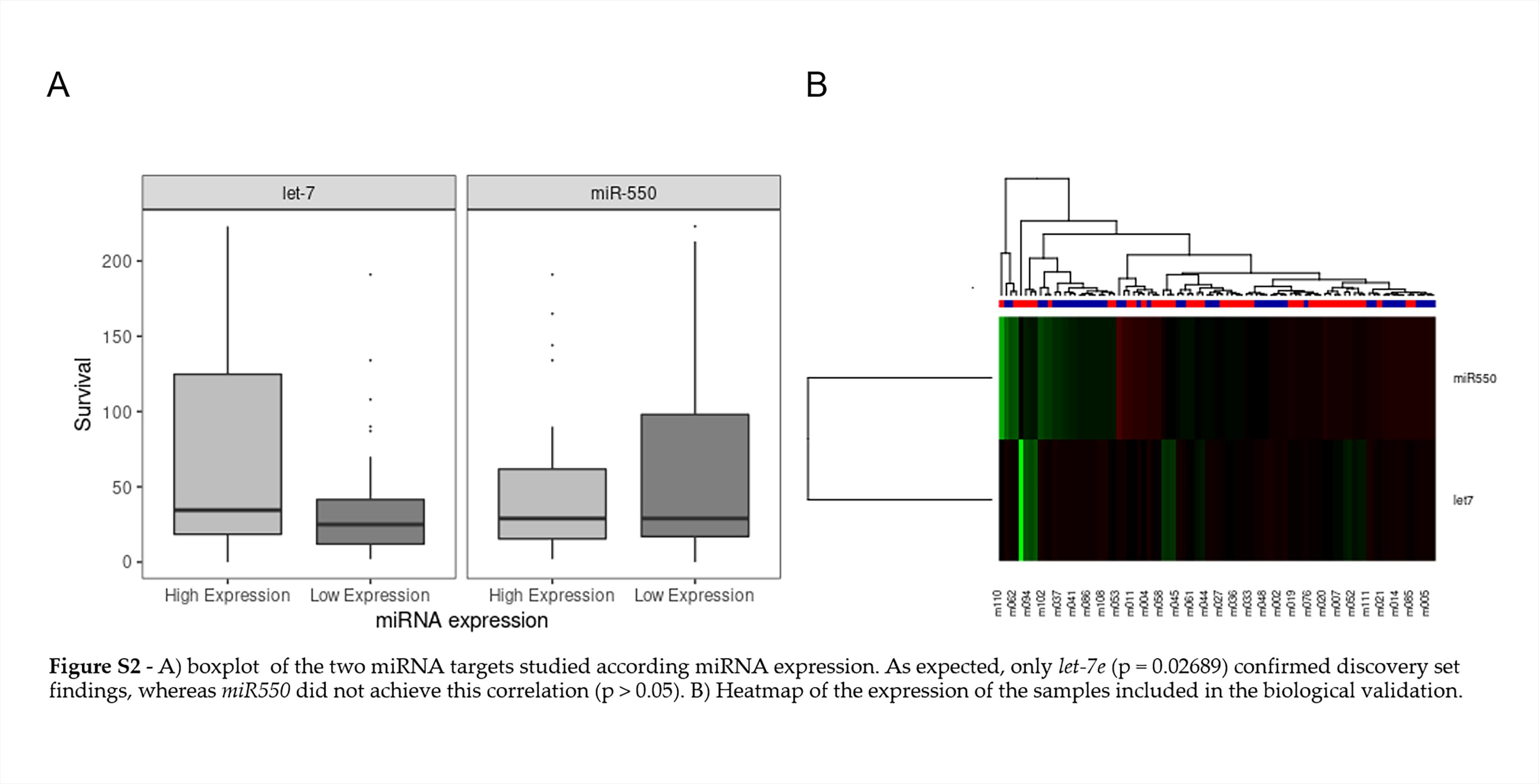

Supplement: Supplementary file 1 [file cancers-12-02979-s001.zip › Figure S2.tif]

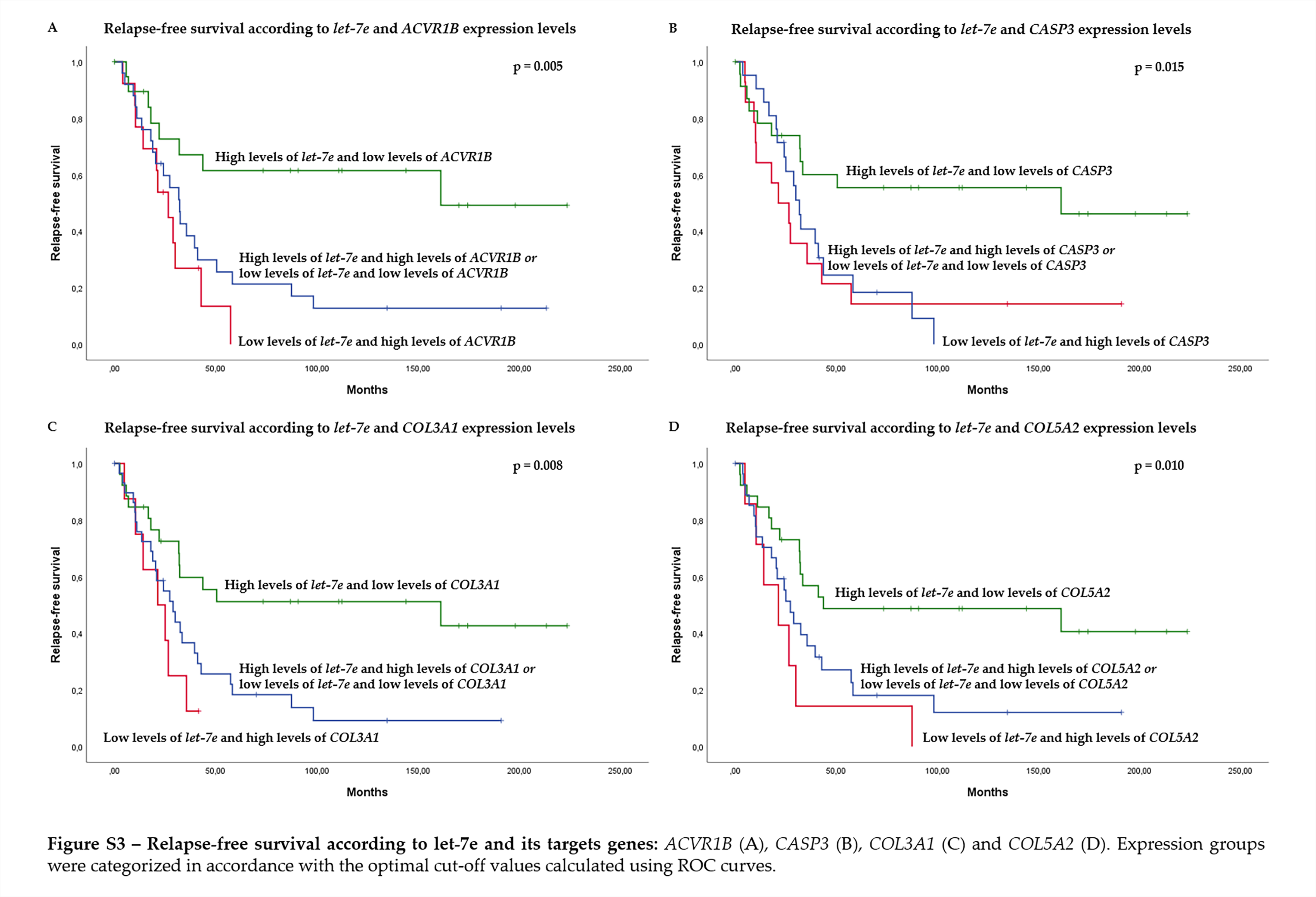

Supplement: Supplementary file 1 [file cancers-12-02979-s001.zip › Figure S3.tif]
